# Intravenous Immunoglobulin Treatment Did Not Improve Tics in a Patient With Gilles de la Tourette Syndrome and Intrathecal Antibody Synthesis

**DOI:** 10.3389/fneur.2020.00110

**Published:** 2020-02-28

**Authors:** Natalia Szejko, Carolin Fremer, Kurt-Wolfram Sühs, Pedro Macul Ferreira de Barros, Kirsten R. Müller-Vahl

**Affiliations:** ^1^Clinic of Psychiatry, Social Psychiatry and Psychotherapy, Hannover Medical School, Hanover, Germany; ^2^Department of Bioethics, Medical University of Warsaw, Warsaw, Poland; ^3^Department of Neurology, Medical University of Warsaw, Warsaw, Poland; ^4^Department of Neurology, Hannover Medical School, Hanover, Germany; ^5^Department of Psychiatry, Institute of Psychiatry, University of São Paulo, São Paulo, Brazil

**Keywords:** Gilles de la Tourette syndrome, intrathecal antibody synthesis, intravenous immunoglobulin treatment, cerebral spinal fluid, oligoclonal bands

## Abstract

Gilles de la Tourette syndrome (GTS) is a neuropsychiatric disorder characterized by motor and vocal tics. There are several hypotheses as to the cause of the disease. One of which suggests that the immune system is involved in the pathophysiology of GTS. Here, we present a 40-year-old female patient with a typical history and clinical presentation of GTS with tics and psychiatric comorbidities, in whom positive oligoclonal bands (OCBs) type 2 in cerebral spinal fluid (CSF) were detected in an earlier study. At that time point, all other investigations were unremarkable (including neurological examination and cMRI), but 2 years later, she presented with further neurological symptoms including tetraparesis mostly affecting the left limbs, distal hypesthesia and paresthesia, and dyspnea. Since further examinations (including EMG, MRI, CSF, virological, and bacteriological tests, as well as autoimmune-encephalitis antibodies) revealed normal results, based on clinical presentation, the diagnosis of Guillain-Barré-like immune-mediated neuropathy was made and treatment with intravenous immunoglobulins (IVIg) (30 g/day for 5 days) was initiated resulting in complete remission of immune-mediated neuropathy. Based on the “immune hypothesis” of GTS, we were interested in whether in this patient positive CSF OCBs might serve as a biomarker for treatment response of tics and additional GTS-related psychiatric symptoms to IVIg, and therefore assessed tics, premonitory urges, and psychiatric comorbidities before and several times after the IVIg treatment. However, even though immune-mediated neuropathy resolved after treatment, tics, premonitory urges, and comorbidities remained unchanged. Thus, this case study suggests that treatment with IVIg is not effective in the treatment of GTS—even in a patient with intrathecal antibody synthesis as expressed by CSF isolated OCBs.

## Case Report

Gilles de la Tourette syndrome (GTS) is a childhood-onset tic disorder often accompanied by different comorbidities such as attention deficit/hyperactivity disorder (ADHD), obsessive-compulsive disorder (OCD), depression, and anxiety. Multiple neurotransmitter systems are thought to be involved in the pathophysiology of GTS including the dopaminergic, GABAergic, serotonergic, glutamatergic, histaminergic, and endocannabinoid systems. In addition, there is certainly a complex interplay of both genetic and environmental factors (1).

With regard to non-genetic influential factors, numerous findings support the hypothesis of the involvement of the immune system in the pathology of GTS. Many studies have investigated the relationship between the occurrence of tics and group A Streptococcus (GAS) infections also known as pediatric autoimmune neuropsychiatric disorders associated with streptococcus infections (PANDAS) (2–4). To shed further light into the proposed association between various environmental factors including GAS infections and different immunological factors and chronic tic disorders including GTS, the large European multicenter study EMTICS was initiated (5). The main conclusion of the EMTICS study is that there are no indications for a causal relation between GAS exposure and the onset or exacerbation of tics and related psychiatric comorbidities such as obsessive-compulsive symptoms (for consulting the final report, see https://cordis.europa.eu/project/rcn/102102/reporting/en?rcn=59137). However, the data provided further evidence for the involvement of immunological factors in the pathogenesis of chronic tic disorders.

Thus, the EMTICS findings are in accordance with several other studies that have consistently shown either an overall activation or distinct abnormalities in the immune system in GTS such as increased levels of cytokines (6, 7) and immunoglobulins (8–10), activation of different types of immune cells (11), and gene expression related to the immune system (12). In line with these data, positive oligoclonal bands (OCB) in cerebral spinal fluid (CSF) have been found in 20–30% of adult patients with GTS indicating an intrathecal antibody synthesis (13, 14). However, until now, no corresponding antibodies could be identified (14). Furthermore, microglia activation was demonstrated both in a postmortem study indicated by increased numbers of CD45+ microglial cells in the striatum (15) and in an imaging study using positron emission tomography (PET) and (11)C-[R]-PK11195 (PK) showing increased binding potential values in the caudate nuclei in children with GTS (16).

Further support for the “immune hypothesis” of GTS comes from studies conducted in animal models and cell cultures based on active immunization with bacterial and viral agents, direct injection of cytokines or a patient's serum antineuronal antibodies, as well as transgenic techniques used to create animal models resulting in the causation of stereotyped behaviors similar to tics [for review, see (17)]. Examples of this causal relationship are as follows: (i) that stereotypic behaviors have been reported in a mouse strain model with altered immune function (BTBR T+tf/J) (18); (ii) in mice treated with interleukin (IL)-6, increased locomotor behavior has been demonstrated (19); (iii) also other immune molecules such as TGF-β1 (20) and soluble IL-2 receptors (sIL-2R) α or β (21) have been found to increase stereotypic behaviors or repetitive movements; and (iv) exposure of male Fischer 344 rats to unfractionated sera or IgG extracted to antibodies from patients with GTS led to motor stereotypies and episodic vocalizations similar to tics (22).

Based on these findings that suggest a dysfunctional immune system in GTS, an immune-based treatment using intravenous immunoglobulins (IVIg) has been proposed as an alternative to current pharmacotherapy with antipsychotics. Until now, available results are limited and highly inconsistent: data from an uncontrolled study (23) in a small number of patients—mostly children with otherwise treatment-resistant GTS—suggest that treatment with IVIg may improve tics, but in contrast, a double-blind placebo-controlled study in 30 unselected patients (aged 16–63 years) failed to demonstrate efficacy (24). However, in another double-blinded study (in 29 children) comparing IVIg not only to placebo but also to plasma exchange, both active interventions were comparably efficacious in the treatment of tics and OCD compared to placebo (25).

Here we present the unique case of a female patient with GTS, who not only exhibited intrathecal antibody synthesis documented by positive OCBs, but in addition received IVIg for the treatment of an unrelated Guillain-Barré-like immune-mediated neuropathy. Based on the hypothesis that positive OCBs might serve as a biomarker not only for an involvement of the immune system in GTS but also for successful treatment of tics and comorbidities, we were interested in whether her GTS-related symptoms might improve after the IVIg treatment.

## Case Description

For the first time, the patient presented in our Tourette outpatient clinic at the age of 40. She was diagnosed with GTS several years before at the age of 5. At the first consultation, she was suffering from multiple motor tics such as head and shoulder shaking, jerks of the whole body, blocking tics that impaired walking, and muscle cramp-like tics, as well as different vocal tics including teeth grinding, deep exhaling, screaming of syllables such as “ha,” and grunting. Further clinical exploration revealed the comorbid diagnoses of OCD, self-injurious behavior, sleeping problems, as well as a history of a depressive episode, alcohol abuse, and benzodiazepine abuse. Treatments with habit reversal training and antipsychotics did not result in a significant improvement of tics or caused intolerable side effects. At age 44, the patient agreed to participate in a study at our clinic that aimed to investigate different CSF parameters in patients with GTS (14). At that time point, she suffered from severe motor tics that provoked pain resulting in a further increase in tics, but further neurological examination and cMRI results were unremarkable and there was no history of any further neurological symptoms or diseases. Her CSF analysis revealed positive OCBs (type 2), while all other immunological investigations were normal.

Two years later, at age 46, she presented in our clinic with a tetraparesis predominantly affecting the left limbs, distal hypesthesia and paresthesia, and dyspnea. Medical history revealed that 3 months before, similar symptoms had occurred for the first time. After admission to an external clinic, lumbar puncture was performed and showed CSF pleocytosis (but no OCBs). Because of this finding, antibiotic treatment was initiated, which resulted in a complete remission of neuropathy-associated symptoms. During hospitalization in our clinic (3 months after the first episode), further examinations revealed normal results for EMG, MRI, CSF, and virological and bacteriological tests, as well as negative results for autoimmune-encephalitis antibodies. Based on the clinical presentation, the diagnosis of Guillain-Barré-like immune-mediated neuropathy was made and treatment with IVIg (30g/day for 5 days) was initiated, which resulted in a complete remission of neuropathy. Another relapse occurred 3 months later, but the patient again responded well to treatment with IVIg (30 g/day for 5 days). At that time, she received no medication for tics or comorbidities.

Because OCBs were found to be positive before the first clinical manifestation of the immune-mediated neuropathy, we were interested in whether her GTS-related symptoms including tics and psychiatric comorbidities might also respond to the IVIg treatment. Therefore, we assessed tics, premonitory urges, and psychiatric symptoms including OCD, ADHD, depression, and anxiety disorder before and several times after treatment with IVIg (results of all clinical assessments are presented in Table 1). However, while the patient recovered from immune-mediated neuropathy, tics and premonitory urges improved only slightly and temporarily 2 weeks after initiation of the IVIg treatment and returned to baseline level thereafter, while psychiatric symptoms remained unchanged.

**Table 1:** Results of clinical assessments before and after treatment with IVIg.

**Symptom/disorder**	**Assessment (range)**	Results during previous CSF study^*^	**Current results**				**Evaluation**
			**BL before IVIg**	**T1**	**T2**	**T3**	
Tics	YGTSS-TTS (0–50)	19	24	19	14	23	Temporary improvement of tics at T2
Premonitory urges	PUTS (9–36)	20	18	13	9	13	Temporary improvement of premonitory urges at T2
OCS	Y-BOCS (0–40)	14	0	–	–	–	No obsessive/compulsive symptoms at BL
Depression	BDI-II (0–63)	4	6	–	–	–	No depression at BL
Anxiety	BAI (0–63)	4	14	–	–	–	Mild anxiety at BL
ADHD	DSM-IV Checklist (0–6)	Inattention: 6 Hyperactivity-Impulsivity: 4	Inattention: 4 Hyperactivity-Impulsivity: 0	–	–	–	No ADHD
Quality of Life	GTS-QoL (0–108)	11	18	0	2	0	Constant improvement after treatment with IVIg (lower scores indicate higher quality of life)

*OCD, obsessive-compulsive disorder; ADHD, attention deficit hyperactivity disorder; YGTSS-TTS, the Yale Global Tic Severity Scale–Total Tic Score; PUTS, Premonitory Urge for Tics Scale; Y-BOCS, the Yale Brown Obsessive Compulsive Scale; BDI, Beck Depression Inventory; BAI, Beck Anxiety Inventory; GTS-QoL, Gilles de la Tourette Syndrome–Quality of Life Scale; BL, Baseline; T1 = 3 days; T2 = 15 days; T3 = 28 days after treatment with IVIg*.

* *24 months before treatment with IVIg*.

## Discussion

Anecdotal reports suggest that IVIg might be effective in the treatment of tics in patients with GTS. Since available data are contradictory, we hypothesized that positive CSF OCBs might serve as a biomarker for successful treatment of tics, premonitory urges, and psychiatric comorbidities using IVIg. We present the unique case of a patient with GTS, in whom positive OCBs had been detected in the context of an earlier study that aimed to investigate immunological factors in GTS (14), and who later developed a Guillain-Barré-like immune-mediated neuropathy. However, while the immune-mediated neuropathy resolved after the IVIg treatment, tics, premonitory urges, and comorbidities remained unchanged. In our opinion, slight and temporary improvement of tics 2 weeks after treatment should be interpreted in the context of the recovery from immune-mediated neuropathy with consecutive reduction of stress and improved mood and anxiety, and subsequent improvement of general well-being and quality of life, but not as a direct treatment effect on tics. Although theoretically conceivable, from our data, it is not suggested that, in this patient, muscle weakness due to immune-mediated neuropathy has influenced the severity of motor tics.

In summary, this case report suggests that treatment with IVIg is not effective in the treatment of GTS—even in patients with intrathecal antibody synthesis as expressed by CSF-isolated OCBs.

## Data Availability Statement

The datasets generated for this study are available on request to the corresponding author.

## Ethics Statement

Written informed consent was obtained from the patient for the publication of this Case Report.

## Author Contributions

KM-V and K-WS conceived and designed the study. KM-V, K-WS, and CF acquired data. CF set up the electronic database. KM-V, K-WS, PM, and NS analyzed and interpreted the data, performed visualization, and reviewed and edited the manuscript. NS wrote the original draft of the manuscript.

### Conflict of Interest

The authors declare that the research was conducted in the absence of any commercial or financial relationships that could be construed as a potential conflict of interest.
